# 

**Published:** 1875-07

**Authors:** 


### Books and Pamphlets Received.

Contributions to the Pathology aud Therapeutics of Diphtheria. By A. Jacobi, M. D., etc. From the Journal of Obstetrics February, 1875.

Irodotomy and its Applicability to Certain Defects of the Eye. By A. W. Calhoun, M. D. From Southern Medical Record.

Analysis of One Thousand Cases of Skin Disease. With Remarks and Cases. By L. Duncan Buckley, M. D. From the American Practitioner, May 1875.

A Clinical Contribution to the Treatment of Tubal Pregnancy. By T. Gaillard Thomas, M. D. From N. Y. Med. Journal, June, 1875.

Annual Announcement of the Detroit Medical College.

Circular and Catalogue of the Savannah Medical College.

Second Annual Announcement of the Hospital College of Medicine, Louisville, Ky.

Annual Announcement of the Medical Department of the University of Buffalo.

